# Investigation of parent-of-origin effects induced by fenofibrate treatment on triglycerides levels

**DOI:** 10.1186/s12863-018-0640-9

**Published:** 2018-09-17

**Authors:** Chloé Sarnowski, Samantha Lent, Josée Dupuis

**Affiliations:** Department of Biostatistics, Boston University School of Public Health, 801 Massachusetts Avenue, Boston, MA 02118 USA

**Keywords:** Triglycerides, Fenofibrate treatment, Epigenetics, Parent-of-origin effects, DNA methylation

## Abstract

**Background:**

Genome-wide association studies performed on triglycerides (TGs) have not accounted for epigenetic mechanisms that may partially explain trait heritability.

**Results:**

Parent-of-origin (POO) effect association analyses using an agnostic approach or a candidate approach were performed for pretreatment TG levels, posttreatment TG levels, and pre- and posttreatment TG-level differences in the real GAW20 family data set. We detected 22 genetic variants with suggestive POO effects with at least 1 phenotype (*P* ≤ 10^− 5^). We evaluated the association of these 22 significant genetic variants showing POO effects with close DNA methylation probes associated with TGs. A total of 18 DNA methylation probes located in the vicinity of the 22 SNPs were associated with at least 1 phenotype and 6 SNP-probe pairs were associated with DNA methylation probes at the nominal level of *P* < 0.05, among which 1 pair presented evidence of POO effect. Our analyses identified a paternal effect of SNP rs301621 on the difference between pre- and posttreatment TG levels (*P* = 1.2 × 10^− 5^). This same SNP showed evidence for a maternal effect on methylation levels of a nearby probe (cg10206250; *P* = 0.01). Using a causal inference test we established that the observed POO effect of rs301621 was not mediated by DNA methylation at cg10206250.

**Conclusions:**

We performed POO effect association analyses of SNPs with TGs, as well as association analyses of SNPs with DNA methylation probes. These analyses, which were followed by a causal inference test, established that the paternal effect at the SNP rs301621 is induced by treatment and is not mediated by methylation level at cg10206250.

## Background

Despite the success of genome-wide association studies (GWAS) conducted for lipid levels, particularly triglyceride (TG) levels, to discover common risk alleles, the genetic factors identified to date only account for a small part of the genetic variance of this trait. Genetic studies have largely focused on DNA sequence variations. Sources of this unexplained heritability might include epigenetic mechanisms such as DNA methylation. Epigenetic modifications are heritable changes influencing gene expression without alterations of the underlying DNA sequence. Few epigenome-wide studies have been conducted for lipid levels, particularly TG levels [1–6]. However, DNA methylation can cause partial or complete silencing of 1 parental allele at a specific locus and can lead to a differential effect of that polymorphism on disease in offspring according to the parental origin of the risk allele (parent-of-origin [POO] effect). Such epigenetic mechanism has been described for lipid levels by Predazzi et al., who reported sex-specific parental effects on offspring lipid levels [7].

Our approach consisted in combining POO effect associations with association of nearby DNA methylation probes with TGs followed by a causal inference test to assess whether methylation mediates the observed POO. This approach has been successful in identifying differentially methylated cytosine-phosphate-guanine (CpG) sites that mediate the association between a genetic variant and a phenotype under a POO effect [8].

**Fig. 1:**
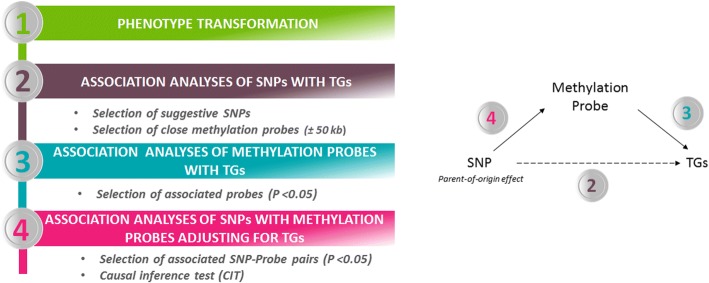
Analytic strategy

We performed POO effect association analyses of pretreatment TG levels, posttreatment TG levels, and pre- and posttreatment TG-level differences in the real GAW20 family data set, using a linear regression approach implemented in the quantitative transmission disequilibrium test (QTDT) software [9]. We conducted 2 approaches to select suggestive POO effect associations: a candidate approach in regions known to be associated with TG levels, and an agnostic approach over the full genome. We then investigated whether the detected POO effects were associated with TG-associated DNA methylation probes and performed causal inference tests. Figure 1 shows the analytical strategy and the biological mechanism of mediation by DNA methylation of the association between the genetic variant and the phenotype (TG).

## Methods

### Triglyceride phenotype transformations

The real data example for GAW20 is from the Genetics of Lipid Lowering Drugs and Diet Network (GOLDN) study data set [4]. This study ascertained and recruited families from the Family Heart Study at 2 centers, Minneapolis, MN and Salt Lake City, UT, who self-reported to be white. TG levels in the GAW20 data set were measured at 4 exams: 2 pretreatment exams (visits 1 and 2) and 2 posttreatment exams (visits 3 and 4). At visit 1, subjects were measured after an overnight fast with a standard lipid profile. The next day, they returned to clinic, again fasting, for a second, repeat lipid profile. All subjects were then given the fenofibrate drug for a 3-week treatment period, after which they returned to the clinic for 2 consecutive days of lipid profiling (visits 3 and 4, both with overnight fasting), to assess the response to treatment. We performed association analyses of single-nucleotide polymorphisms (SNPs) with the average natural-log TG pretreatment (TG_1–2_), the average natural-log TG posttreatment (TG_3–4_), and the natural-log TG difference between pre- and posttreatment (∆TG_1–4_) using a linear mixed-effect model to account for familial correlations and adjusted for age, sex, center (0 = Minnesota, 1 = Utah), and smoking (coded as never, former, or current).

### Association analysis of SNPs with TG level

GWAS genotyping was performed on DNA extracted from blood drawn at baseline, using the Affymetrix Genome-wide Human SNP Array 6.0. Association analyses were conducted using the QTDT software with and without accounting for POO effects. From the real data, we analyzed 823 genotyped individuals from 173 families (715,787 SNPs). We evaluated the association of principal components calculated in the GAW20 data set with the traits evaluated in this study. As population stratification was not expected to be a major concern, we used the QTDT *-at* option to perform a total association analysis [9]. This model is not a transmission disequilibrium test and has a considerably higher power and greater efficiency to detect POO effects. The trait mean of child *j* in family *i* is modeled as: *Y*_*ij*_ *= α + β*_*G*_*G*_*ij*_ *+ e*_*ij*_ where *β*_*G*_ is the genetic effect of the minor allele (*a*) of the SNP and *G*_*ij*_ is the number of minor alleles carried by child *j* in family *i*.

Then, our association analyses focused on POO effects on TG in the offspring. This approach involves modeling the phenotype as a function of covariates and genotypes, taking parental original of each allele into consideration [10]. We use the QTDT with POO effect options (*−of*, *−ot*, *−om*, and *−op*) to get estimates of parental origin of the alleles [9]. The model for the mean of the trait of child *j* in family *i* is: *Y*_*ij*_ *= α + β*_*mat*_ *G*_*1ij*_ *+ β*_*pat*_ *G*_*2ij*_ *+ e*_*ij*_ where *β*_*mat*_ is the genetic effect of a maternally inherited minor allele, *β*_*pat*_ is the genetic effect of a paternally minor allele, *G*_*1ij*_ is the estimated number of minor allele inherited from the mother, and *G*_*2ij*_ is the estimated number of minor alleles inherited from the father. The *-at -ot* tests for difference between paternal and maternal transmissions (*β*_*pat*_ *= β*_*mat*_), the *-at -of* uses a saturated model where maternal and paternal inherited alleles are modeled separately (*β*_*pat*_ *= β*_*mat*_ *= 0*), whereas the *-at -om* and *-at -op* tests for maternal effect (*β*_*mat*_ *= 0*) or paternal effect (*β*_*pat*_ *= 0*) only. We modified the QTDT to test 1 parental effect while adjusting for the other parent contribution (*om_op* and *op_om* models).

Selection of suggestive POO effects was performed using 2 different approaches. We first used a candidate approach in regions known to be associated with TG levels using the GWAS catalog (http://www.ebi.ac.uk/gwas/search?query=triglycerides). We selected 14 European-ancestry GWAS after excluding GWAS performed on specific phenotypes (responses to treatment) or related phenotypes (obesity, Type 2 diabetes, lipids). A total of 129 variants in 59 regions were reported associated with TG levels at *P* < 10^− 5^ (threshold to include associations in the GWAS catalog). We used a ± 50-kb window around the reported SNPs to define candidate loci. We used an arbitrary threshold of 10^− 5^ to declare a SNP association suggestive under the POO effect model (in at least one of the following models: *ot*, *of*, *om*, *op*, *om_op*, *op_om*). We then used an agnostic approach over the full genome with the same arbitrary threshold of 10^− 5^ to declare a SNP association suggestive under the POO effect model (in at least 1 of the following models: *ot*, *of*, *om*, *op*, *om_op*, *op_om*).

### Association analyses of DNA methylation probes with TGs

DNA was extracted from CD4+ T cells and the proportion of sample methylation at > 450,000 CpG sites was quantified by using the Illumina Infinium Human Methylation 450 K BeadChip from blood drawn in both visit 2 and visit 4 for each participant. We selected DNA methylation probes located within a ± 50-kb window around the suggestive SNPs under the POO effect model. We performed epigenetic association analyses of natural-log TG pretreatment (TG_2_), natural-log TG posttreatment (TG_4_), and natural-log pre- and posttreatment TG difference (∆TG_2–4_) using a linear mixed-effect model adjusted for age, sex, center, and smoking to account for familial correlations. From the real data, we analyzed 679 and 403 individuals from 163 and 136 families with genotypes and methylation available at exam 2 or 4, respectively. We selected all DNA methylation probes associated with at least 1 trait at the nominal level of *P* < 0.05.

### Association analyses of SNPs with methylation probes

Association analysis between DNA methylation probes and SNPs was conducted using the same approach described for SNP-TG association, using the QTDT software to evaluate the association between suggestive SNPs under the POO effect model and significant DNA methylation probes, while including the phenotype in the model.

### Causal inference test

We performed a causal inference test [11] to determine if the detected POO effects were mediated by DNA methylation at nearby probes associated with the phenotypes. The causal inference test comprises 4 conditions that have to be met to conclude that mediation is responsible for the observed association: the SNP and the phenotype are associated; the SNP is associated with methylation after adjusting for the phenotype; the methylation is associated with the phenotype after adjusting for the SNP; and the SNP is independent of the phenotype after adjusting for the methylation [11].

## Results

Using an agnostic approach on the genome, we identified 19 SNPs with a suggestive POO effect (*P* < 10^− 5^), among which 6 were associated with TG_1–2_, 1 with TG_3–4_, and 12 with ∆TG_1–4_. Among the SNPs previously reported to be associated with TG level, only 8 were associated with the phenotypes (TG_1–2_) at *P* ≤ 10^− 3^ and no SNP showed strong POO effects (*P* > 10^− 3^). Using a candidate approach, we identified 2 SNPs with a suggestive POO effect (*P* < 10^− 5^), among which 2 were associated with TG_1–2_ (rs7559813, *P op_om* = 8.3 × 10^− 6^ and rs10417097, *P op_om* = 8.0 × 10^− 6^) and 1 was also associated with TG_3–4_ (rs10417097, *P op_om* = 8.5 × 10^− 7^). We identified 18 DNA methylation probes located in the vicinity of these SNPs that were associated with the phenotypes at *P* < 0.05 (11 with TG_2_, 3 with TG_4_, and 4 with ∆TG_2–4_). We detected 6 SNP-probe pairs with association (P < 0.05) in at least 1 model with QTDT (Table 1), among which 1 pair (rs301621–cg10206250) presented evidence of POO effect. We identified a paternal effect of rs301621 on chromosome 16 on pre- and posttreatment TG difference, ∆TG_1–4_ (*P op_om* = 1.2 × 10^− 5^) and detected a maternal effect of the same SNP on methylation levels of a nearby probe (cg10206250, *P om_op* = 0.01). The observed paternal effect of rs301621 remains significant when adjusting for DNA methylation at cg10206250 (*P op_om* = 5.5 × 10^− 5^) and is also associated with posttreatment TG (*P op_om* = 0.001).

**Table 1:** Association results between SNPs associated with TGs (TG_1–2_or TG_3–4_ or ΔTG_1–4_) under the POO model and nearby DNA methylation probes associated with TGs (TG_2_ or ΔTG_2–4_), with or without taking the POO effect into account

SNP	Chr:Pos (Mb)	Gene	Pheno	Alleles a1/a2	Freq a1	TGs = SNP + covariates					TGs = Meth_2_ + covariates			Meth_2_ = SNP + covariates + TGs				
						P	P of^†^	P ot^†^	P om_op^†^	P op_om^†^	Probe	Pos (Mb)	P	P	P of^†^	P ot^†^	P om_op^†^	P op_om^†^
rs2903839	2:56.17		TG_1–2_	A/G	0.59	0.16	2.7 × 10^−7^	1.5 × 10^− 7^	1.8 × 10^− 6^	0.01	cg07468585	56.2	0.02	0.53	0.02	0.39	0.21	0.01
											cg25249448	56.2	0.03	0.55	0.04	0.16	0.62	0.01
rs13131918	4:24.87	*CCDC149*	TG_1–2_	A/G	0.52	0.07	2.8 × 10^−7^	1.4 × 10^−7^	0.004	7.0 × 10^−7^	cg09490880	24.9	0.04	0.02	0.72	0.52	0.42	0.85
rs7300117	12:131.7		TG_1–2_	A/T	0.52	6.2 × 10^−9^	3.8 × 10^−5^	0.07	0.11	3.9 × 10^− 5^	cg09481537	131.7	0.04	0.09	0.06	0.16	0.70	0.02
rs301621	16:86.65		ΔTG_1–4_	G/T	0.60	0.18	1.5 × 10^−5^	0.01	0.40	1.2 × 10^− 5^	cg10206250	86.6	0.04	0.19	0.02	0.008	0.01	0.10
rs10417097*	19:19.72	*PBX4*	TG_1–2_	C/G	0.62	0.01	3.0 × 10^−5^	6.4 × 10^−5^	0.23	8.0 × 10^−6^	cg10738025	19.7	0.03	0.005	0.47	0.92	0.34	0.49
rs10417097*	19:19:72	*PBX4*	TG_3–4_	C/G	0.62	0.009	3.0 × 10^−6^	6.8 × 10^−6^	0.13	8.5 × 10^−7^	–	–	–	–	–	–	–	–

*SNP located in a locus previously reported to be associated with TGs (rs17216525-T, beta = 0.11, *P* = 4.0 × 10^− 11^)

^†^Statistical significances of: (a) the test when maternal and paternal inherited alleles are modeled separately (*β*_*pat*_ *= β*_*mat*_ *= 0*, *P of*), (b) the test for difference between paternal and maternal transmissions (*β*_*pat*_ *= β*_*mat*,_ *P ot*), (c) the test of maternal effect only (*β*_*mat*_ *= 0*, *P om_op*), and (d) the test of paternal effect only (*β*_*pat*_ *= 0*, *P op_om*)

## Discussion

We detected 3 SNPs with POO effect associations that reached the multiple-testing correcting threshold using the agnostic approach (*P* ≤ 5 × 10^− 8^) or the candidate approach threshold (*P* ≤ 1.2 × 10^− 5^). The tested methylation probes were only associated with the tested phenotypes before multiple testing correction. This could be from a lack of power because of the small number of individuals analyzed. However, we detected 19 SNPs with suggestive POO effects (*P* < 10^− 5^) and this may reflect new mechanisms of action of these SNPs, particularly in previously reported loci such as SNP rs10417097 on chromosome 19 in the *PBX4* gene [12, 13] with a strong paternal effect on both TG_1–2_ and TG_3–4_ (*P* ≤ 10^− 5^) and associated with methylation levels of cg10738025 (*P* = 0.005, see Table 1). Only one-third of the SNPs previously reported to be associated with TGs were available in the GAW20 data. These SNPs did not present strong POO effects (*P* > 10^− 3^) and few of them were strongly associated with the phenotypes at P ≤ 10^− 3^. This could be from a lack of power because of the small number of individuals analyzed.

## Conclusions

We performed association analyses in the real GAW20 data set to identify POO effects on TG levels in offspring. These analyses were followed by association analyses of SNPs with DNA methylation probes adjusting for TG levels. Interestingly, the G allele of rs301621 was associated with pre- and posttreatment TG difference when transmitted by the father and with methylation levels of cg10206250 when transmitted by the mother. We showed that this paternal effect is induced by treatment. Using a causal inference test, we demonstrated that the observed POO effect of rs301621 on the difference between pre- and posttreatment TG level was not mediated by DNA methylation at cg10206250. Further investigation in a larger number of participants is needed to confirm the POO effects identified in this report.

## Abbreviations

CpG: Cytosine-phosphate-guanine
GOLDN: Genetics of lipid lowering drugs and diet network
GWAS: Genome-wide association studies
POO: Parent-of-origin
QTDT: Quantitative transmission disequilibrium test
SNP: Single nucleotide polymorphism
TG: Triglyceride
